# Clinical Diagnoses and Characterization of Patients With Amyloid-Negative Amyloid-Beta, p-Tau, and Neurofilament Light Chain (ATN) Profiles

**DOI:** 10.7759/cureus.75874

**Published:** 2024-12-17

**Authors:** Colin Barnett, Kiel Morris, Yogesh Shah

**Affiliations:** 1 College of Osteopathic Medicine, Des Moines University, West Des Moines, USA; 2 Geriatric and Memory Center, Broadlawns Medical Center, Des Moines, USA

**Keywords:** alzheimer's disease, amyloid negative, atn, biomarkers, dementia, geriatrics, mild cognitive impairment, neurocognitive disorder, non-alzheimer's, subjective cognitive impairment

## Abstract

The novel amyloid-beta, p-Tau, and neurofilament light chain (ATN) classification scheme has become a promising system for clinically detecting and diagnosing Alzheimer's disease (AD). In addition to its utility in Alzheimer's diagnosis and treatment, the ATN framework may also have clinical relevance in identifying non-Alzheimer's pathologies. In this study conducted at Broadlawns Geriatric and Memory Center, 92 amyloid-negative profiles out of 182 patients with an ATN framework were categorized into subjective cognitive impairment (SCI), non-amnestic mild cognitive impairment (non-amnestic MCI), amnestic MCI, Alzheimer's dementia, vascular dementia, mixed dementia, unspecified dementia, or other memory changes based on diagnoses written in the chart. Additionally, other secondary diagnoses were found in the differential, including sleep disorders, anxiety, depressive disorders and grief, and cerebrovascular disease. The results are concordant with our expectations that amyloid-negative ATN profiles are associated with mostly non-Alzheimer's cognitive decline. We were also able to demonstrate that amyloid-negative patients have other secondary neurologic or psychiatric diagnoses related to memory or cognitive changes. However, certain enigmatic patient presentations warrant further scrutiny in the medical chart. It is possible that ATN may pose a risk of misclassification in both Alzheimer and non-Alzheimer pathologies, particularly at early stages. Future work may be required to corroborate findings using other new plasma biomarkers, such as p-Tau217. Overall, we hope that this study will provide options for early detection and future treatment of AD and other neurocognitive disorders. We also anticipate that this work will lead to the recognition of other non-neurocognitive conditions comorbid with such neurocognitive disorders.

## Introduction

The novel amyloid-beta, p-Tau, and neurofilament light chain (ATN) classification scheme has become a promising system for detecting and diagnosing Alzheimer's disease (AD) in vivo [1-5]. The standardization of this approach allows for the identification of plasma ATN biomarkers indicative of AD [6]. These blood-based biomarkers (BBMs) for amyloid pathology are increasingly sensitive and specific, albeit more variable than in cerebrospinal fluid [7-9]. It is evident that ATN testing has diagnostic and prognostic utility [9,10]. Patients with pre-clinical or enigmatically mild decline may have distinct ATN profiles suggesting an increased risk of further decline toward major neurocognitive disorder (MNCD), otherwise known as "dementia" [6,11-15]. Therefore, ATN testing adds substantial diagnostic value to non-biomarker testing, e.g., cognitive screening tests such as the Mini Mental Status Examination (MMSE), especially in early or enigmatic cases [16-18]. Such enigmatic cases may include amyloid-negative ATN profiles, which may indicate other forms of memory or neurocognitive changes [3,13,19,20]. Moreover, non-Alzheimer's neurodegenerative pathologies may also feature abnormal ATN profiles, although research in this area is still limited [3,21]. Thus, further effort is needed to characterize a clinical diagnosis of patients with non-Alzheimer and amyloid-negative ATN profiles.

Furthermore, other comorbidities may pose a risk of cognitive decline and feature with detectable signs of neurodegeneration [3], e.g., conditions (such as depression) that have been associated with an increased risk of dementia [14]. ATN may differentiate whether certain conditions independent of degenerative disease are comorbid or prodromal [14,22-24].

This work analyzes the diagnostic implications and correlations of amyloid-negative ATN profiles with clinical diagnosis, stage, and secondary comorbidities. We hope to corroborate and expand upon the findings of these BBMs to inform the utility of ATN testing in clinical practice.

## Materials and methods

This study was conducted at Broadlawns Geriatric and Memory Center where ATN testing is routinely used in new evaluations, utilizing a robust electronic medical record (EMR) system. This project was given institutional review board (IRB) exempt status, as determined by the Institutional Review Board at Des Moines University. Broadlawns Medical Center has an arrangement with Des Moines University's Institutional Review Board for all IRB activities conducted by medical students. Additionally, two of Broadlawns' senior staff hold positions in the Des Moines University IRB. The ATN tests were performed at a laboratory citing 96% sensitivity and ~87% specificity for Alzheimer's disease, according to Labcorp [25]. The primary data points included amyloid (A), tau (T), and neurofilament light chain (N) values, stage of decline, clinical cognitive diagnoses, and non-dementia comorbidities. Inclusion criteria were all patients seen at Broadlawns Geriatric and Memory Center from January 18, 2024, to June 6, 2024, who received plasma biomarker ATN (A for amyloid, T for tau, and N for neurodegeneration) and were negative for A (amyloid). Exclusion criteria were amyloid-positive ATN profiles, as well as other charts that were initially compiled for ATN testing but had missing or otherwise incomplete ATN data (n=90). There were no exclusion criteria based on race, gender, age, or other demographic attributes. The chart review included 182 patients who presented with cognitive concerns and had an existing ATN profile, which included 92 patients with amyloid-negative results (A-T-N-, A-T+N-, A-T-N+, and A-T+N+).

**Figure 1:**
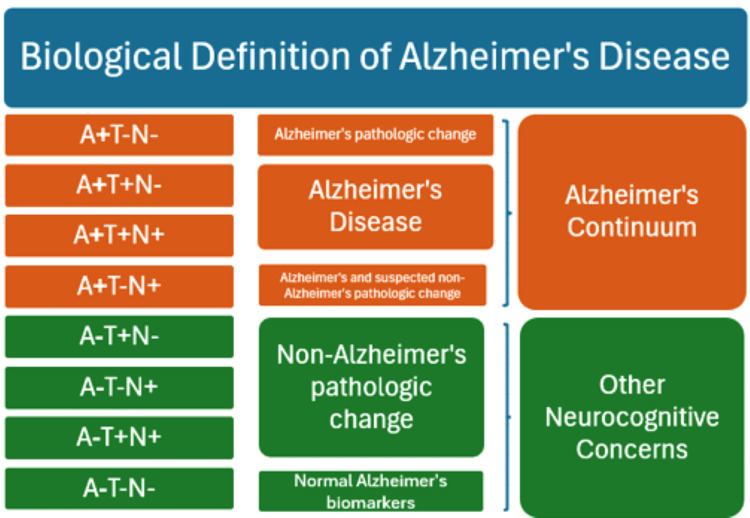
Schematic of ATN profiles and classification of Alzheimer's and non-Alzheimer's pathologies Note: Amyloid-positive (A+) profiles designate pathologies along the Alzheimer's continuum, while amyloid-negative (A-) profiles may still indicate other neurocognitive concerns not related to Alzheimer's disease. A: amyloid, T: tau, N: neurodegeneration

Stages of decline are defined as subjective cognitive impairment (SCI), amnestic mild cognitive impairment (MCI), non-amnestic MCI, and major neurocognitive disorder. Clinical neurodegenerative diagnoses identified included "Alzheimer's disease", "vascular", "mixed pathology", "neurocognitive disorder unspecified", and "other memory changes". These stages of decline and clinical neurodegenerative diagnoses were classified based on the specific terms used in the electronic medical records. ATN profiles were then compared to the neurocognitive diagnosis to determine alignment with Alzheimer's and non-Alzheimer's classifications, based on a modified version of a schematic by Jack et al. (Figure 1) [3]. SCI refers to self-reported cognitive decline not reported on cognitive testing, while MCI refers to an initial manifestation of dementia without significant decline in daily living function.

Among non-neurodegenerative diagnoses, 76 relevant secondary comorbidities were identified and, for practical purposes, were further categorized as anxiety, depression and grief, sleep disorders, cerebrovascular disease, substance/alcohol use, bipolar disorders, attention deficit disorder/attention deficit hyperactivity disorder (ADD/ADHD), autism spectrum disorder (ASD), intellectual disability, post-traumatic stress disorder (PTSD), hearing loss, and polypharmacy/medication side effects.

## Results

**Figure 2:**
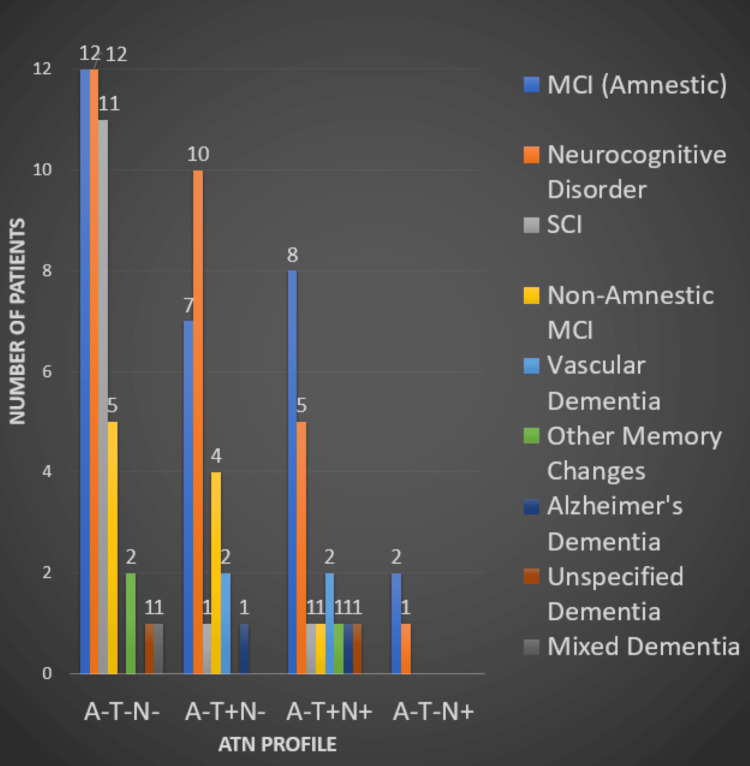
ATN profile and neurocognitive diagnoses Note: This bar graph shows the frequencies of ATN profiles and our classification of neurocognitive diagnoses for the 92 amyloid-negative patients. A: amyloid, T: tau, N: neurodegeneration, MCI: mild cognitive impairment, SCI: subjective cognitive impairment

Of the 92 patients, 44 were A-T-N-, 25 were A-T+N-, 20 were A-T+N+, and three were A-T-N+. In the A-T-N- group, 11 (25%) were classified as subjective cognitive impairment, 12 (27%) were classified as amnestic MCI, five (11%) were classified as non-amnestic MCI, 12 (27%) were classified as neurocognitive disorder unspecified, two (4.5%) were classified as other memory changes, one (2.3%) was classified as mixed dementia, and one (2.3%) was classified as unspecified dementia. In the A-T+N- profiles, 10 (40%) were classified as neurocognitive disorder, seven (28%) were classified as amnestic MCI, four (16%) were classified as non-amnestic MCI, two (8%) were classified as vascular dementia, one (4%) was classified as SCI, and one (4%) was classified as Alzheimer's dementia. In the A-T+N+ group, eight (40%) were classified as amnestic MCI, five (25%) were classified as neurocognitive disorder, two (10%) were classified as vascular dementia, one (5%) was classified as SCI, one (5%) was classified as non-amnestic MCI, one (5%) was classified as Alzheimer's dementia, one (5%) was classified as unspecified dementia, and one (5%) was classified as other memory changes. Lastly, in the A-T-N+ profiles, two (66%) were classified as amnestic MCI and one (33%) was classified as neurocognitive disorder (Figure 2). Overall, we found that vascular dementia (n=32), amnestic MCI (n=29), and SCI (n=13) were the most common neurocognitive diagnoses found in the amyloid-negative patient sample. It is also worth noting that 12 (13%) out of 92 amyloid-negative patients had an A-T-N- profile and a diagnosis of "Neurocognitive Disorder" or "Major Neurocognitive Disorder" written in the chart.

**Figure 3:**
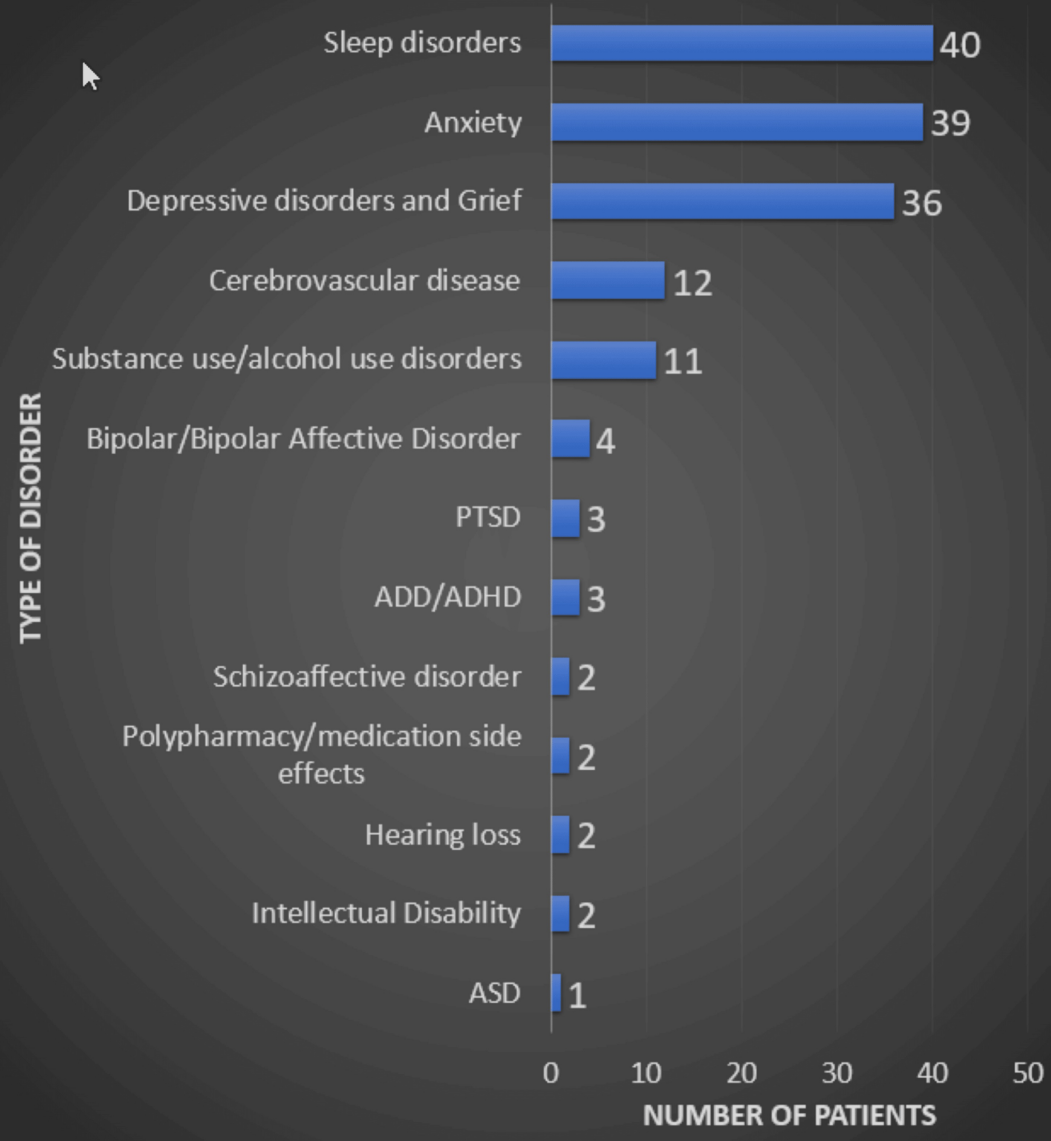
Most common secondary comorbidities Note:* *This bar graph shows the frequency of secondary comorbidities associated with amyloid-negative ATN profiles. A: amyloid, T: tau, N: neurodegeneration, PTSD: post-traumatic stress disorder, ADD/ADHD: attention deficit disorder/attention deficit hyperactivity disorder, ASD: autism spectrum disorder

In addition to the neurocognitive diagnoses, we found that the most common clinical secondary comorbidities were sleep disorders (n=40), anxiety (n=39), depressive disorders and grief (n=36), cerebrovascular disease (n=12), substance/alcohol use disorders (n=11), bipolar/bipolar affective disorder (n=4), PTSD (n=3), intellectual disability (n=2), ADD/ADHD (n=3), schizoaffective disorder (n=2), polypharmacy/medication side effects (n=2), hearing loss (n=2), and ASD (n=1) (Figure 3).

## Discussion

Our findings align with our hypothesis that amyloid-negative ATN profiles are primarily associated with non-Alzheimer's etiologies and other cognitive concerns. We observed that amyloid-negative patients frequently had secondary diagnoses related to memory or cognitive changes. However, the presence of multiple comorbid diagnoses in patient charts limits our ability to draw definitive conclusions about individual contributions.

Counterintuitively, 13% of patients classified as having "Neurocognitive Disorder" with an amyloid-negative ATN profile, who should not meet the criteria for a dementia diagnosis, suggest potential false-negative results from the ATN test. These enigmatic cases warrant further investigation and follow-up for differential diagnoses. While speculative, ATN may misclassify both Alzheimer's and non-Alzheimer's pathologies, especially in the early stages. Future studies using p-Tau217 biomarkers, a more refined diagnostic tool, may help confirm these findings [26].

The variability in documentation formats among patient charts made initial diagnosis classification challenging. Many terms used in the electronic medical record appeared convoluted and interchangeable. For instance, "Neurocognitive Disorder" was used quite often, as was "Major Neurocognitive Disorder", but the distinction between such similar terms was categorized together. This study may have limitations in accurately categorizing patient diagnoses. Our work could inform the development of more standardized and concise charting approaches for future clinical practice.

Patient sampling limitations include the study population being drawn from individuals already presenting with memory concerns and the selection of patients based on clinician discretion for ATN testing. These factors may have introduced bias into the data. Further research is needed to analyze a more diverse patient population. Due to the study's methodology, descriptive and comparative analyses, such as chi-square tests, were limited. Statistical analyses could provide a more comprehensive characterization of the data. Additionally, exploring ATN profiles across different stages of Alzheimer's disease, from subtle cognitive impairment to severe dementia, would be valuable. However, ATN biomarkers are limited in that they are an indirect measure of pathologic brain changes [1]. Moreover, changes in ATN biomarkers are evident years before cognitive changes [5].

With the emergence of new plasma biomarker combinations for Alzheimer's disease, such as ATN, cognitive decline can be predicted 4-5 years earlier in cognitively intact individuals [27]. The predictive power of combined plasma biomarkers is superior to individual biomarkers [28]. Therefore, the ATN model remains a promising tool for diagnosing AD and other forms of cognitive decline in the near future [29].

There are limitations of blood-based biomarkers. Biomarkers are less sensitive than neuropathology for the detection of mild/early pathology. Validated biomarkers are not available for all relevant neuropathologies of dementia; therefore, it cannot be known with certainty in vivo what neuropathologies in addition to AD are present in any individual or what the proportional neuropathologic burden is among various pathologies. Biomarkers should not be used in isolation but should always be interpreted in a clinical context [30].

## Conclusions

This study of 92 amyloid-negative patients examined neurocognitive diagnoses and secondary clinical diagnoses in relation to the ATN framework. This work leads to a more harmonized understanding of amyloid-negative ATN profiles consistent with patient presentations with cognitive concerns. While some exceptions were observed, our findings generally support the congruence between these diagnoses and the ATN model. Moreover, it underscores the utility and importance of blood-based biomarkers for dementia and other memory/neurocognitive concerns in clinical practice.
